# Molecular tools of antibiotic resistance for *Helicobacter pylori*: an overview in Latin America

**DOI:** 10.3389/fgstr.2024.1410816

**Published:** 2024-07-15

**Authors:** Monica Contreras, Heisser Mujica, María Alexandra García-Amado

**Affiliations:** Laboratorio de Fisiología Gastrointestinal, Centro de Biofísica y Bioquímica, Instituto Venezolano de Investigaciones Científicas (IVIC), Altos de Pipe, Venezuela

**Keywords:** antibiotic resistance, molecular methods, genotyping, Helicobacter pylori, Latin America

## Abstract

Antibiotic resistance in the treatment of *H. pylori* infection is the principal reason for the failure of eradication regimens using the triple therapy. We reviewed the mechanisms of *H. pylori* antibiotic resistance and assessed the molecular tools commonly used to detect antibiotic resistance in Latin America. Most commonly reported molecular techniques were PCR and sequencing, as well as its variants PCR-RFLP and qPCR to detect the genes and mutations involved in the resistance to clarithromycin (CLR), amoxicillin (AMX), levofloxacin (LVX), tetracycline (TET), and metronidazole (MTZ). The mutation-associated resistance rates varied from 2.2% to 83.3% for CLA and 12% to 100% for LVX in 7 and 4 countries, respectively, whereas for MTZ the resistance frequency was between 50% to 100% in 4 countries. For TET and AMX, frequency of the resistance was in the range of 0% to 27% (5 and 3 countries, respectively). Molecular tools can be used for the rapid detection of resistance to CLR, LVX, and TET, whereas genotypic analysis is not appropriate to detect resistance to AMX and MTZ due to genomic changes found in the *rdxA* and *pbp1A* genes. The revision of the different molecular methods showed that qPCR and RFLP are the best tools to detect the resistance of *H. pylori*. Few molecular tools have been developed in Latin America to detect *H. pylori* resistance to antibiotics. More studies would be needed to understand better efficient strategies to prevent further emergence of antimicrobial resistance.

## Introduction

Antimicrobial resistance is a significant public health problem worldwide, characterized mainly by multidrug-resistant pathogen bacteria (1). In the last years, global antimicrobial utilization increased by at least 35.0% in most countries (2). *Helicobacter pylori (H. pylori)* is among the most prevalent global pathogens, colonizing an estimated 50.0% of the world’s population (3). Its prevalence ranges from 40.0% to 50.0% in developed countries and up to 90.0% in developing countries (3, 4). The emergence of antibiotic-resistant strains is a primary cause of treatment failure in *H. pylori* infections. The detection of antibiotic resistance by phenotypic methods such as agar diffusion (E-test) or agar dilution has the advantage that resistance to the same antibiotic is observed by diverse mechanisms (5). However, these methods require up to two weeks for completion, and occasionally fail due to either a lack of growth of *H. pylori* or overgrowth of other bacteria (6). Therefore, molecular tools are increasingly used for the detection of single nucleotide polymorphisms in genes associated with *H. pylori* resistance to multiple antibiotics within a few days (7). It has already been shown that molecular tools are excellent for the prediction of antibiotic resistance and it would define novel therapeutic schemes for *H. pylori* eradication (7). This review discusses the molecular tools reported in Latin America for antibiotic resistance in *H. pylori*.

## Literature search strategy

A literature search was performed PubMed, Scielo, and Google Scholar databases. Articles published from 1990 to July 2023, were screened using the following keywords: *H. pylori* antibiotic resistance, Latin American countries, in combination with ‘detection’ and/or ‘molecular’. We selected articles that evaluated molecular tools used for the detection of antibiotic resistance in *H. pylori* and *gyrA/gyrB*, 16S *rRNA*, 23S *rRNA*, *rdxA*/*frxA*, and *pbp1A* genes. Based on these criteria we found published research performed in nine countries such as Argentina, Brazil, Chile, Colombia, Cuba, Ecuador, Mexico, Peru, and Venezuela.

## Treatment schemes

Different treatment schemes have been recommended among the regimens used in the treatment of *H. pylori* infection including a combination of two antibiotics and a proton-pump inhibitor (PPI) (Triple Therapy, TT) or conjoin of these agents with bismuth salts (bismuth-based quadruple therapy, BQT) for 14 days or 10 days (8, 9). The TT regimen is commonly used in the first-line treatment for *H. pylori*-infected patients (PPI plus clarithromycin (CLR), amoxicillin (AMX), or metronidazole (MTZ)) (9). This scheme is suitable in populations with CLR resistance rates lower than 15.0%, and without prior exposure to CLR (10). If a failure of a first-line treatment that includes CLR (triple or quadruple) occurs, a therapy of second-line treatment with levofloxacin (LVX), preferably quadruple (PPI, AMX, LVX, and bismuth) is recommended. An alternative as a third line is a concomitant quadruple treatment (PPI, AMX, CLR, and nitroimidazole). Should there be a failure of a third treatment, rifabutin therapy represents an encouraging strategy to prescribe a fourth line for a period of only 10 days, in this case (PPI, AMX, and rifabutin) (10).

In Latin America, there is no consensus own for the treatment of *H. pylori* infection. Most countries follow the guidelines of the Maastricht consensus report, which includes antibiotics with high resistance (e.g. CLR and MTZ), as it has been reported worldwide. Therefore, the Maastricht VI/Florence Consensus Report (2022) recommended not using CLR and MTZ treatments in case of simultaneous resistance to both antibiotics greater than 15.0% (9, 11). This suggests the need to have appropriate surveillance programs, study genetic changes given to drug resistance, and monitor its evolution to improve antimicrobials and increase public awareness (7, 12).

## Clarithromycin (CLR)

CLR is a macrolide antibiotic that binds to the 50S unit of the bacterial ribosome (13). Many studies have shown that the CLR resistance mechanism of *H. pylori* is associated with mutations in the 23S *rRNA* gene domain V and alter the binding of CLR to the peptidyl-transferase region (14, 15). The resistance to CLR is associated with three main point mutations at positions A2142G or A2142C and A2143G at the 23S *rRNA* gene that block the CLR binding site at the 50S bacterial ribosomal subunit (16–18). The A2142C, A2142G, and A2143G mutations were reported for the first time in *H. pylori* strains isolated from Canada and the United States, respectively (17, 18). Resistance to CLR has been studied worldwide and its prevalence varies in each country depending on the seropositivity rate (19).

The *H. pylori* CLR resistance in clinical samples has been reported by different molecular methods, such as restriction fragment length polymorphism (RFLP), real-time PCR or quantitative PCR (qPCR), Random Amplified Polymorphic DNA (RAPD), DNA sequencing, PCR line probe assay (PCR-LiPA) and other PCR variants (**Table 1**). All these tools have been shown can detect *H. pylori* CLR resistance with excellent specificity and sensitivity on DNA of different samples (gastric biopsies, gastric juice, and stool) if culture is not possible (36, 37).

**Table 1:** Studies of antibiotics resistance-associated mutations of *H. pylori* in American Latin.

Antibiotics	Country	Phenotype methods/ % resistance rates	Molecular methods used/ % resistance rates*	Mutations detected in the 23S rRNA, *pbp1A*, *gyrA/gyrB*, 16S rRNA and *rdxA/frxA* genes (%)**	Total from *H. pylori*-positive specimens	References
CLR	Argentina	Agar dilution / 26.9%	RAPD-PCR and sequencing / 76.0%	A2143G (89.5%), A2142G (10.5%) and other mutations (A2267G or T1861C) not related to CLR resistance	197 isolates	(5)
	Brazil	E-test / 26.7%	qPCR / 83.3%	A2142G and A2143G (83%)	31 stool DNAs	(20)
	Brazil	–	qPCR / 14.4%	A2142G (37.5%), A2143G (62.5%) and A2142G+A2143G (12.5%)	222 gastric mucosa and juice DNAs	(21)
	Chile	–	qPCR and sequencing / 31.2%	A2142G (31.0%) and A2143G (69.0%)	93 gastric mucosa DNAs	(22)
	Chile	–	PCR-RFLP / 26%	A2142G (10.5%) and A2143G (89.5%)	69 gastric mucosa DNAs	(23)
	Colombia	Agar dilution / 46.0%	PCR and sequencing / 44.6%	C2196T (0.05-0.20%), T2183C (0.09-0.20%), A2144G (0.25%), A1593T, A1653G, C1770T, C1954T and G1827C (30.0%)	74 isolates	(14)
	Colombia	–	PCR and sequencing / 38.1%	A2143G (22.2%) and A2142G (7.9%)	126 isolates	(16)
	Colombia	Agar dilution / 50.0%	PCR and sequencing / 50.0%	A2143G (80%)	10 isolates	(11)
	Colombia	–	Sequencing / 3.6%	A2142G (83.3%) and A2143G (16.7%)	166 whole genome sequences	(7)
	Colombia	–	PCR-RFLP / 18.8%	A2143G (81.5%), A2142G (11.1%) and A2143G+A2142G (7.4%)	143 gastric mucosa DNAs	(24)
	Colombia	Agar dilution / 39.2%	ASP-PCR / 39.2%	A2142G (9.5%) and A2143G (90.5%)	107 isolates	(25)
	Ecuador	–	qPCR and sequencing / 33.2%	A2142G (27.9%), A2143G (11.3%) and A2142G+A2143G (60.8%)	238 gastric mucosa DNAs	(26)
	Mexico	Disk-diffusion / 17.8%	PCR-RFLP / 2.2%	A2143G (2.2%)	45 isolates	(27)
	Peru	Broth microdilution / 52.3%	PCR-RFLP / 43.5%	A2142G (30.5%) and A2143G (13.0%)	44 isolates	(28)
AMX	Argentina	Agar dilution / 7.6%	RAPD-PCR and sequencing / 10.0%	Thr556Ser (40%), Ser543Arg (40%), Glu406Ala, Ser417Thr, Thr556Ser and Asn562Thr (20%) in resistant isolates and Ser543His in one sensitive isolate	197 isolates	(5)
	Colombia	Agar dilution / 20.0%	PCR and sequencing / 20.0%	Glu406Val (50.0%)	10 isolates	(11)
	Colombia	–	Sequencing / 25.9%	Arg649Lys (41.9%), Thr593Ser, Thr593Ala and Thr593Pro (30.2%), Arg656Pro+Arg656His (18.6%) and Thr556Ser (9.3%)	166 whole genome sequences	(7)
	Ecuador	–	qPCR and sequencing / 6.7%	Multiple amino acid substitutions: Asn504Asp (12.5%), Asn562Tyr (6.25%), Val374Leu (18.75%), Gly595Ser (6.25%), Asp479Glu (12.5%), Ala474Thr+Asp479Glu (6.25%), Thr593Ala (6.25%), Asn504Asp+Asn562Asp (12.5%), Thr593Gly (6.25%), Thr593Ser (6.25%) and Asn504Asp, Asp479Glu, Thr593Ser (6.25%) not specific in resistant strains	238 gastric mucosa DNAs	(26)
LVX	Argentina	Agar dilution / 34.6%	RAPD-PCR and sequencing / 92.8%	Asn87Ile, Asn87Lys, Asp91Gly, Asp91Asn and Asp91Tyr (92.8%) in *gyrA*	197 isolates	(5)
	Colombia	Agar dilution / 100.0%	PCR and sequencing / 100%	Asn87Ile (50.0%), Asp91Asn (25.0%) and Asn87Lys (25.0%) in *gyrA*	10 isolates	(11)
	Colombia	Agar dilution / 18.0%	PCR and sequencing / 18.2%	Asn87Ile (43.8%), Asp91Gly (28.8%), Asn87Lys (11.3%), Asp91Asn (5.0%), Asn87Tyr (1.3%), Asp91Try (1.3%), Asn87Tyr+Asp91Gly (1.3%) and Asn87Ile+Asp91Gly (1.3%) in *gyrA*	439 gastric mucosa DNAs	(29)
	Colombia	–	Sequencing / 12.0%	Asn87Lys+Asn87Ile (45%); Asp91Gly, Asp91Asn and Asp91Tyr (55%) in *gyrA*	166 whole genome sequences	(7)
	Colombia	Agar dilution / 48.6%	ASP-PCR / 52.3%	Asn87Ile (44.6%), Asn87Lys (12.5%), Asp91Gly (35.7%), Asp91Asn (5.4%) and Asn87Tyr+Asp91Gly (1.8%) in *gyrA*	107 isolates	(25)
	Ecuador	–	qPCR and sequencing / 39.9%	Asn87Lys (28.4%), Asp91Asn (17.9%), Asp91Gly (17.9%), Asp91Tyr (3.2%), Asn87Lys+Asp91Tyr (1.1%), Asn87Ile (12.5%), Asn87Thr (8.4%), Asn87Asp (6.3%), Asn87Asp+Asp91Asn (2.1%), Asn87Phe (1.1%) and Asn87Thr+Asp91Asn (1.1%) in *gyrA*	238 gastric mucosa DNAs	(26)
	Venezuela	E-test / 47.0%	PCR and sequencing / 80.0%	Asn87Thr or Asn87Ile (62.5%) in *gyrA* and Asn87Thr+Asp91Asn*+*Ser479Gly (37.5%) in *gyrA* and *gyrB*	60 isolates	(30)
TET	Brazil	–	PCR-RFLP / 0%	AGA926–928TTC (0%)	395 gastric mucosa DNAs	(31)
	Chile	Agar dilution / 26.8%	PCR-RFLP / 26.8%	A928C (9.1%), AG926–927GT (54.5%) and AGA926–928GGC (36.4%)	41 isolates	(32)
	Colombia	–	Sequencing / 7.2%	A926G or A926T (75.0%) and A928C (25.0%)	166 whole genome sequences	(7)
	Ecuador	–	qPCR and sequencing / 4.2%	AGA926–928GGA (60.0%), AGA926–928GTA (20.0%), AGA926–928AGC (10.0%), AGA926–928AGT (10.0%) and AGA926–928TTC (0%)	238 gastric mucosa DNAs	(26)
	Venezuela	E-test / 14.6%	qPCR / 14.0%	AGA926–928GGA (23.1%)	96 isolates	(33)
	Chile	–	PCR and sequencing / 50.0%	Multiple substitutions in *rdxA*: Arg16His, Ala67Val, Ser88Pro and Gly162Arg (57,2%), Glu75Gln (7.1%), Pro166Ala (7.1%) Pro166Ser (7.1%), 2 truncating mutations (14.3%) and 1 an in-frame deletion (7.1%)	28 gastric mucosa DNAs	(22)
MTZ	Colombia	–	PCR and sequencing / 78.2%	Multiple substitutions in *rdxA*: Asp59Asn (90%), Arg131Lys (59.4%), Arg90Lys (57.1%), Ala118Thr (24.7%), Ile160Phe (18.8%), His97Thr (15.3%) and other single mutations and stop codons (21.4%)	170 gastric mucosa DNAs	(34)
	Colombia	Agar dilution / 70.0%	PCR and sequencing /70.0%	Arg90Lys (28.6%) in *rdxA*	10 isolates	(11)
	Colombia	–	Sequencing / 99.3%	Multiple substitutions in *rdxA*: Asp59Asn (99.3%), Arg131Lys (47.3%), Arg90Lys (46.7%), His97Thr, His97Tyr and His97Ile (26.1%), Ala118Thr, Ala118Ser and truncations (24.8%) and a stop codon	166 whole genome sequences	(7)
	Cuba	–	PCR and sequencing / 56.8%	Multiple substitutions in *frxA:* mutations producing stop codons of high level (27.9%), single mutations of less level (48.6%) and news stop codons (16.2%)	37 isolates	(35)
	Ecuador	–	qPCR and sequencing / 100%	Multiple amino acid substitutions in *rdxA*: Asp59Asn (100%), Arg90Lys (52.5%), Arg131Lys (56.7%), and other in minor proportions as Met56Ile, Arg16His, Gln50Stop, Leu62Val, Ala118Thr, Met56Val, Gly98Ser, His97Thr, Ser88Pro, His97Tyr, Arg16Cys, Ala68Val and Ala183Val	238 gastric mucosa DNAs	(26)

(*) percentage resistance rates per molecular tool were calculated by dividing the number of samples with mutations between the number of *H. pylori*-positive samples and multiplying by 100.

(**) percentage of detected mutation distribution was calculated by dividing the number of specific mutation samples between the number total of resistance samples reported in the previous column (*) or frequency of mutation found and multiplying by 100. Antibiotics: CLR, Clarithromycin; AMX, Amoxicillin; LVX, Levofloxacin; TET, Tetracycline; MTZ, Metronidazole.

Using RFLP, with restriction enzymes *BbsI* and *BsaI*, CLR resistance in Colombia was determined by A2143G and A2142G mutations in 18.8% (27/143) of *H. pylori*-positive samples when the A2142G mutation is detected, digestion of the amplicons of the peptidyltransferase region of the 23S *rRNA* gene with the *BbsI* enzyme generates two fragments of approximately 93 and 332 base pairs, whereas the *BsaI* enzyme produces three fragments of 20, 300, and 105 bp in A2143G mutation (GenBank U27270) (24). The most frequent mutation was A2143G 81.5% (22/27) (24). These results demonstrate a high CLR resistance rate, as previously reported in Colombia (12). In a study performed in Mexico, using PCR-RFLP, on 63 *H. pylori*-positive isolates, mutation A2143G in domain V of the 23S *rRNA* gene was the only one found in eight (1/8) phenotypically CLR-resistant isolates (12.5%) (27). However, other point mutations (A1821G; G1826A; T1830C; A2089G; T1600C; C1601T; C1602T; T1610C; A1611C and T1633G), which have not been associated with CLR resistance, were identified (27). It is possible that these mutations are related to CLR resistance or that resistance is conferred by alternative mechanisms like efflux pumps, methylases presence, or plasmids carrying antibiotic resistance genes (27). This study reported the largest proportion of resistant *H. pylori* strains not harboring the A2142G, A2142C, and A2143G mutations in the 23S *rRNA* gene (87.5%) (27). Another study in Peru on 95 patients also used PCR-RFLP to determine mutations associated with CLR resistance. The resistance to CLR was observed in 43.5% and specific A2142G and A2143G mutations were detected in 30.5% and 13.0% respectively, demonstrating that CLR resistance rate and percentage of *H. pylori* strains circulating were high (28).

One of the molecular tools most encouraging is the qPCR probe hybridization technology using fluorescence resonance energy transfer (FRET) probes, which can rapidly detect CLR resistance in biopsies and isolates with mixed infections of clinical specimens (37). Moreover, this tool is easy, rapid, and low cost; qPCR is technically achievable in developing countries (37). Therefore, the qPCR assay allows the localization of single mutations associated with CLR resistance, and the TaqMan probes provide improving melt resolution, enabling the prediction of susceptibility to CLR (38). A study in Brazil validated the biprobe qPCR stool assay from 217 dyspeptic children (20). Forty-five patients (20.7%) were *H. pylori*-positive by qPCR assay in biopsy samples, whereas 31 of them were found to be positive in stool samples. The CLR resistance was determined by PCR in 10 of 12 stool DNA specimens, associated with mutations A2142G or A2143G (83.3%). This qPCR assay in stool was demonstrated to be useful for the assessment of CLR resistance in *H. pylori* (20). In addition, a study from the Northeastern of Brazil, evaluated also resistance to CLR by qPCR in 14.4% (32/222) of *H. pylori*-positive samples (21). The A2142G and A2143G single mutations were found in 37.5% (12/32) and 62.5% (20/32) respectively, and double mutations (A2142G plus A2143G) in 12.5% (4/32) (21). The qPCR has been used to detect CLR resistance in different sample types, both in the gastric mucosa and juice and in feces, compared to culture (21).

Studies performed in Chile, Colombia and Ecuador determined mutations associated with CLR resistance, using the PCR amplification and sequencing of the 23S *rRNA* gene domain V of *H. pylori* (7, 11, 22, 23, 26). In Ecuador, 238 DNA samples (without and with unsuccessful prior *H. pylori* eradication treatment) were analyzed and the mutation-associated CLR resistance rate was 33.2% in *H. pylori*-positive samples (26). This study showed differences between patients without and with unsuccessful treatment, and single and multiple mutations such as A2142G (27.9%), A2143G (11.3%), and A2142G+A2143G (60.8%), demonstrating a high *H. pylori* resistance rate to CLR (26). Similarly in Chile, CLR resistance prevalence was 31.2% in 29 biopsies from *H. pylori*-positive gastric mucosa using a 5’exonuclease qPCR assay and sequencing. The A2142G and A2143G mutations were detected in 9 (31.0%) and 20 (69.0%) samples, respectively (22). CLR resistance showed heteroresistance of strains in the same patient, with 16 homoresistant strains and 13 heteroresistant strains. The authors concluded that the prevalence of CLR resistance needs to be re-evaluated in the *H. pylori* treatment in Chile (22). A CLR resistance prevalence was also observed in 19 (26.0%) out of 69 *H. pylori*-positive patients in an independent Chilean study (23). The A2143G and A2142G mutations were found in 89.5% (17/19) and 10.5% (2/19) of samples, respectively. Whereas in a meta-analysis of previous studies in Chile, pooled CLR resistance prevalence was higher at 31.3%, showing heterogeneity low between the three studies evaluated. This result confirms the high CLR resistance in Santiago City, Chile, and its association with the failure of *H. pylori* treatment (23). Interestingly, another study by Guzman et al. (7) identified mutations in specific genes associated with CLR resistance in 166 *H. pylori* whole genome sequences from Colombia (7). The CLR resistance rate of *H. pylori* was 3.62% (6/166) associated with mutations A2142G in 83,3% (5/6) and A2143G in 16,7% (1/6) of isolates (7). Although these mutations are the most frequent, they do not pass Colombia’s established resistance rate threshold. Indeed, 55 of out 166 sequences were found multidrug-resistant isolates, and all isolates belong to a specific *H. pylori* lineage (hspColombia) in the Andean region from Colombia (7). Comparable results among different studies from Latin America showed that the proportion of single mutations (A2142G and A2143G) associated with CLR resistance varies in frequency among molecular tools used (5, 16, 25).

The Random Amplified Polymorphic DNA (RAPD)-PCR amplification, in cases of resistance and heteroresistance, was detected and characterized in *H. pylori* single and/or mixed infections from Colombia (14, 16). In the study by Matta et al. (14), the amplification and sequencing of 23S *rRNA* gene domain V of *H. pylori* (fragment of 662 bp) was carried out in 56 (76.0%) of 74 isolates from two populations from Colombia (Tumaco and Túquerres). Of 56 isolates studied, 25 (44.6%) were resistant and 31 (55.4%) susceptible to CLR under *in vitro* conditions. In 17 of 25 resistant isolates (13 from Tumaco and 4 from Túquerres) single mutations were found in each population, A1593T1, A1653G2, C1770T, C1954T1, and G1827C in isolates from Tumaco, and A2144G from Túquerres, whereas T2183C and C2196T mutations in both populations. The mutations T2183C, A2144G, and C2196T were reported for the first time in Colombia (14). However, there was no significant association between the *H. pylori* mutations and *in vitro* CLR resistance. These results on the presence of single mutations outside the amplified region (between nucleotides 1585 and 2247) may reflect high genotypic variability among the isolates. The authors concluded that failure of treatment in this Colombian population was associated with *H. pylori* CLR-resistant single mutations (14). Arévalo-Jaimes et al. (16) used the RAPD-PCR analysis to identify infections with single and/or mixed strains in Colombia. This study included 126 isolates of the gastric mucosa (antrum and corpus biopsies) from 63 symptomatic patients, positive for *H. pylori* culture. Overall, the prevalence of *H. pylori* isolates resistant to CLR was 38.1% (24/63 patients). Of these, 19 patients had resistant isolates in both stomach biopsies, 14 with A2143G mutation (22.0%) and 5 with A2142G mutation (7.9%), whereas 5 patients had heteroresistance. DNA fingerprinting analysis of heteroresistant and resistant samples showed that most patients were infected with a single strain except for four with different patterns of mixed infection (16). It is important to highlight the usefulness of molecular tools for the characterization of *H. pylori* infection, especially to identify heteroresistant isolates (16). In Colombia, *H. pylori* CLR resistance studies using antimicrobial phenotypic tests reported a prevalence lower than 20.0% (24, 39, 40), whereas, by DNA sequencing of PCR products a higher prevalence of 38.1% was found (16). A similar prevalence of CLR resistance (39.2%) was reported in a study in Bogotá using Allele-specific PCR (AS-PCR) (25). These results in Colombia may suggest that *H. pylori* resistance to CLR has increased over the years as it is happening worldwide. An additional study in Argentina used the RAPD-PCR in 197 isolates obtained from 52 *H. pylori*-positive patients without previous eradication therapy showed distinguishable banding patterns (5). Fingerprint similarity between 15.0 to 75.0% was found in all isolates from a single individual, except for six of them, which harbored isolates with slightly different profiles. These results strongly indicate that *H. pylori* CLR resistance changes from preexisting susceptible strains rather than co-infections with different strains (5).

A worldwide multicenter study (Australia, Belgium, Brazil, France, Netherlands, and Switzerland) tested 299 *H. pylori* strains (41). Mutations in the 23S *rRNA* associated with CLR resistance were analyzed by PCR and PCR-line probe assay (LiPA). Of the 299 strains, 129 (43.1%) contained single mutations (A2143G (44.1%) and A2142G (32.6%)), and 38 (19.8%) carried multiple 23S *rRNA* mutations. Minimal Inhibitory Concentrations (MICs) of CLR for the A2142G mutant strains were significantly higher than MICs for the A2143G strains. These results show that PCR-LiPA permits a reliable detection of CLR resistance in *H. pylori* (41).

Novel molecular techniques such as Peptide Nucleic Acid (PNA) probes for Fluorescence *in situ* hybridization (FISH), Amplification Refractory Mutation System (ARMS) with qPCR (ARMS‐qPCR), and Peptide Nucleic Acid (PNA)-based qPCR have been developed in different countries as Portugal (42), China (15), and South Korea (43). These techniques combine the detection and *H. pylori* CLR resistance, showing that A2142G and/or A2143G mutations were perfectly concordant with conventional PCR and sequencing analysis. This concordance suggests that these methods are of significant interest to determining CLR resistance in the clinical field to improve *H. pylori* treatment (15, 42, 43). However, these molecular tools have not been yet reported in Latin America.

In Latin America (Argentina, Cuba, Ecuador, Brazil, Chile, Colombia, Costa Rica, Honduras, Mexico, Peru, and Venezuela), the prevalence of CLR resistance varies from 2.0 to 46.0% according to used phenotypic antimicrobial test, as agar dilution (AD), disk diffusion (DD) and epsilon test (E-test) (1, 4, 5, 14, 20, 25, 27, 28, 40, 44). On the other hand, using molecular tools, resistance rate to CLR is higher, ranging from 18.8 reaching 83.3%, in Argentina, Brazil, Chile, Colombia, Ecuador, and Peru; and lower from 2.2% to 14.4% in Brazil, Colombia, and Mexico, as shown in **Table 1**. When comparing the results obtained in 7 studies between the molecular tools and phenotypic tests, a concordant CLR resistance frequency could be seen in both methods for 4 studies (11, 14, 25, 28). In 2 studies, the frequency of CLR resistance was higher in molecular tools than in phenotypic tests due to mutations at other positions that did not confer resistance (5, 20), however, in a study the percentage (2.2%) was lower by molecular tools than phenotypic results. This difference was given by other resistance mechanisms than the authors did not evaluate (27). It is important to mention that although CLR is the antibiotic with the most resistance studies reported by molecular tools in Latin America, there are variable differences between the reports in the range of 2.2 to 83.3% (**Table 1**). In general, CLR resistance is increasing worldwide, reported similar ranges in Asia (15, 43, 45), Europe and USA (38, 41, 42, 46, 47). The development of CLR resistance has been seen as favored due to the breach of treatment and their use in the treatment of respiratory infections (24, 48).

## Amoxicillin (AMX)

AMX interferes with peptidoglycan synthesis by binding to penicillin-binding proteins (PBPs) (49). The PBPs are enzymes with a transpeptidase in the C-terminal region, located on the surface of the cellular membrane allowing the β lactam ring contained in the AMX to block the synthesis of peptidoglycan of the bacterial wall. Multiple substitutions in the transpeptidase C-terminal region are necessary for the expression of AMX resistance in *H. pylori* (49–51). *H. pylori* AMX resistance has been associated with changes in the PBPs–PBP1 (encoded by the *pbp1A* gene), PBP2, and PBP3. The PBP1 and PBP3 mutations have been associated with a higher AMX resistance or those only in PBP1 (51). The resistance has also been associated with a PBP-D absence (tolerance to AMX), combined amino acid substitutions in the porins HopB and HopC, and a reported TEM-1 β-lactamase (36, 52, 53). Gerrits et al. (47) demonstrated that AMX resistance is caused by various mutational changes in the gene encoding the PBP1A protein. In *H. pylori* the Ser402Gly, Glu406Ala, Ser414Arg, Ser417Thr, Thr555Ser, and Asn561Tyr substitutions represent the main factors in the resistance (49). The 69A/AMX^R^ strain has been analyzed and it showed four *pbp1* mutations (Sert414Arg, Tyr484Cys, Thr541Ile, and Pro600Thr) and one *pbp2* mutation (Thr498Ile). All these mutations cause amino acid changes in the AMX^S^ strains 26695, J99, and 69A. The transformation with the mutated *pbp1* gene from the 69A/AMX^R^ strain rendered sensible strains a moderate AMX resistance (MIC of 0.5 to 1 mg/ml). Transformation with the *pbp2* gene from 69A/AMX^R^ caused no AMX resistance. The co-transformation of *pbp1* and *pbp2* did not show increased resistance compared with *pbp1* alone (54). Mutations in *pbp1* have been shown to affect level resistance to AMX of 69A/AMX^R^ strain. This indicates that mutations in more than one gene are probably required to render *H. pylori* AMX^R^ strains (54). The rapid detection of AMX resistance in *H. pylori* by molecular tools is difficult because of the large variety of PBP1A mutations (49). However, previously studies have demonstrated that only a few amino acid substitutions in PBP1 can modify AMX binding-Ser414Arg; Thr438Met; Phe473Leu; Ser543Arg; Thr556Ser; and Asn562Tyr (49, 50, 52–58). Only three of these substitutions (Ser414Arg; Thr556Ser; and Asn562Tyr) have been reported in multiple clinical isolates, suggesting that these are the most common amino acid changes in PBP1 associated with AMX resistance (58).

Zerbetto De Palma et al. (5) investigated point mutations by PCR and sequencing of the *pbp1A* gene, and natural transformation assays determined its association with AMX resistance in 197 *H. pylori*-resistant isolates from Buenos Aires city (5). AMX resistance rate was 7.6% by agar dilution method and 5.0% by PCR and sequencing. The PBP1A amino acid variations in the AMX-resistant isolates and several susceptible ones were similar to those previously reported in β-lactam resistance strains (Glu406Ala, Ser417Thr, Ser543Arg, Thr556Ser, and Asn562Tyr) and isogenic susceptible isolates (Ser543His) (5, 56). Transformation demonstrated that Asn562Tyr and/or Thr556Ser substitutions in the PBP1A confer the AMX resistance in the isolates by amino acid changes. In addition, Ser414Arg substitution was absent in the isolates of this study; however, it has been found in AMX-resistant isolates in different geographic areas (5). Another study in Ecuador, multiple mutations (Asn504Asp, Asn562Tyr, Val374Leu, Gly595Ser, Asp479Glu, Asn504Asp, Ala474Thr, Thr593Ala, Asn562Asp, Thr593Gly, and Thr593Ser) were detected in resistant strains, but only Asn562Tyr mutation was found in resistant isolates from Argentina (5, 26). Currently, the detailed mechanism underlying AMX resistance in *H*. *pylori* is unclear (26). In Colombia, a study with ten *H. pylori* isolates from five patients was conducted to investigate antibiotic resistance to AMX conjoined to other antibiotics by agar dilution, and confirmed by amplification and sequencing the *pbp1A* gene (11). According to the phenotypic sensitivity analysis, two isolates of *H. pylori* AMX resistant were obtained from a single patient (20.0%). The mutation associated with AMX resistance was Glu406Ala (10.0%), confirmed by sequencing in one of two isolates, and eight isolates were resistant to two or more antibiotics (11). Similarly, another study in Colombia (7), using 166 *H. pylori* whole genome sequences found AMX resistance in 25.9% (43/166), with variable mutations in the *pbp1A* gene, including Arg649Lys 41.9% (18/43), Thr593Ser, Thr593Ala, and Thr593Pro 30.2% (13/43), Arg656Pro and Arg656His 18.6% (8/43) and Thr556Ser 9.3% (4/43). The high AMX resistance has been also reported in another region from Colombia where only one substitution in the *pbp1A* gene was found (11).

Variable resistance rates for AMX in the range of 0% to 38.0% were reported in different countries from Latin America (Peru, Honduras, Brazil, Chile, Mexico, Argentina, Colombia, and Venezuela) applying mainly broth microdilution, E-test, and agar dilution (1, 4, 5, 11, 28, 40, 44). Similarly, using PCR and sequencing-based detection AMX resistance rates from 5.0% to 25.9% were found in four studies conducted in Argentina, Colombia, and Ecuador (**Table 1**). In 2 studies, the molecular tools and phenotypic tests were compared, and frequency results were similar in both methods. However, the molecular techniques are not recommended for AMX resistance detection due to multiple mutations in *pbp1A* that are not specific to resistant strains, indicating that it should be complemented with phenotypic tests. These resistance results in Latin America were according to those reported by whole genome sequencing (WGS) in the USA (7.0%) (59), molecular pathologic detection in China (9.0%) (60), and PCR and sequencing in Egypt (18.8%) (61).

## Levofloxacin (LVX)

LVX is a fluoroquinolone of high activity against Gram-negative and Gram-positive bacteria, inhibiting the mechanism of action of the DNA gyrase and topoisomerase IV (62, 63). LVX is an effective alternative to CLR in TT (62). However, quinolone resistance in *H. pylori* was increasing range from 18.0% up to >30.0% and could weaken its efficacy (29, 64).

The common mechanism for fluoroquinolone resistance comprises point mutations in the Quinolone Resistance-Determining Region (QRDR) of the *gyrA* and *gyrB* genes (63). The QRDR of the *gyrA* gene that codes for the subunit A of DNA gyrase, includes codons 86, 87, 88, and 91 (36, 63, 65). The main substitutions have been reported at positions 87 (Asn to Lys) and 91 (Asp to Gly, Asp to Asn, or Asp to Tyr) of the *gyrA* gene (30, 66–68). Therefore, a single mutation in the *gyrA* gene results in a fluoroquinolone-resistant phenotype from the gastric and esophageal mucosa (29, 30). The subunit B of the DNA gyrase presents the amino acid substitutions at positions 435 (Asp to Asn), 463 (Glu to Lys), and 482 (Ile to Met) (66). Studies suggest that mutation at position 463 of *gyrB* could explain the existence of resistant isolates without mutations in *gyrA* (29, 66, 69). However, the *gyrB* mutations do not often happen and have little influence on primary LVX resistance (66).

Molecular tools to detect the single mutations have the potential advantage of providing rapid results to antibiotic resistance (25). In Latin America, numerous authors have investigated single mutations in the *gyrA* and *gyrB* genes of LVX-resistant *H. pylori* isolates and gastric mucosa biopsies, and more recently from the esophageal mucosa through PCR and DNA sequencing, Allele-specific (ASP)-PCR and Random Amplified Polymorphic DNA (RAPD)-PCR (**Table 1**).

Using PCR and *H. pylori gyrA* and *gyrB* sequencing have determined the prevalence and changes associated with primary LVX resistance in Colombia, Ecuador, and Venezuela (4, 28, 62, 64). Trespalacios-Rangél et al. (29) found a LVX resistance rate of 18.2% (80 of 439 samples) according to the range reported in Colombia during the study period (2009 to 2014) from 11.8% (12/102) in 2009 to 27.3% (21/77) in 2014. The most prevalent mutation was Asn87Ile (43.8%, 35/80) followed by Asp91Asn (28.8%, 23/80) and Asn87Lys (11.3%, 9/80), and others in lower proportion. These mutations were detected in the *gyrA* gene in 94% (75/80) of patients with resistant strains, as the principal mechanism of LVX resistance (29). In addition, a descriptive study carried out in Colombia evaluated the resistance to LVX and other antibiotics through agar dilution and DNA sequencing in five patients who had received more three failed treatments for *H. pylori* (11, 29). Eighty percent (8/10) of isolates were resistant to LVX with three different changes (Asn87Ile, Asp91Gly, and Asn87Lys) in the *gyrA* gene and showed resistance to two or more antibiotics, indicating that multi-resistance could be a consequence of failed treatments or acquired resistance by prolonged consumption of antibiotics (11, 70). The results of this study demonstrate the multiple *H. pylori* resistance in Colombia in previously treated patients, with LVX resistance rate greater than the reported in untreated patients, and highlight the importance of implementing sensitivity tests, either by culture or molecular tools, before a first treatment for *H. pylori* to guide first-line therapy (11). Recently, another study in Colombia identified also single mutations in the *gyrA* gene; finding a resistance rate of 12.04% (20/166) to LVX (7). Of the 20 resistant isolates, 55% (11/20) isolates showed the mutations Asp91Gly, Asp91Asn, and Asp91Tyr (50%), and 45% (9/20) had the mutations Asn87Lys and Asn87Ile. These mutations have been related to therapeutic failure of *H*. *pylori* infection (7). In Venezuela, 47% (28/60) of all isolates from the gastroesophageal mucosa of patients were resistant to LVX. In the gastric mucosa, several isolates showed single mutation at position 87 (Asn87Thr or Asn87Ile) or double mutations at positions 87 (Asn87Thr) and 91 (Asp91Asn) of the *gyrA* gene (30). In the esophagus, the isolates showed the same single and double mutations at positions 87 and 91, as those found in the gastric mucosa (30). The mutation Asn87Ile, has only been reported in South Africa, Senegal (65), and Asia (China, Nepal, and Malaysia) (67, 71–73). Studies in Colombia and Argentina corroborated that these mutations confer resistance to antibiotics, depending on the geographical origin (5, 22, 29, 30). Likewise, in the analysis of the *gyrB* gene, amino acid changes at position Ser479Gly (30, 66). In both mucosae, isolates from two patients presented the same nucleotide changes for *gyrA* and *gyrB* genes. This suggests that *H. pylori* isolates in the esophageal mucosa come from the stomach (30). Additionally, a total of 238 DNA samples were analyzed by qPCR in a study from Ecuador (26). A high LVX resistance rate was found at 39.7% (mean). The main mutation in the *gyrA* gene associated with resistance to LVX was Asn87Lys (28.4%), followed by different amino acid exchanges in *gyrA* such as Asn87Ile (12.6%), Asp91Asn (17.9%), Asp91Gly (17.9%), and other changes were found in lower proportion (26). Similar results have been reported in strains LVX resistant in Argentina, Colombia, and Venezuela (5, 29, 30).

Allele-specific PCR (AS-PCR) was used to identify *H. pylori* 23S *rRNA* (to CLR) and *gyrA* (to LVX) mutations using gastric biopsies from Colombian patients and confirmed by PCR and sequencing of the 23S *rRNA* and *gyrA* genes. In 107 *H. pylori*-positive biopsies, AS-PCR found 56 resistant (52.0%) for *gyrA* gene, showing different single mutations as Asn87Ile (25/56), Asn87Lys (7/56), Asp91Gly (20/56), Asp91Asn (3/56), and one double mutation Asn87Tyr and Asp91Gly (1/56) (22). The AS-PCR provides a specific and rapid tool to determine single nucleotide polymorphism in DNA samples (25, 63).

Random Amplified Polymorphic DNA (RAPD)-PCR genotyping tool was used also to investigate the single mutations in the *gyrA* and *gyrB* genes of 197 *H. pylori* primary resistant isolates from Buenos Aires City (5). A high LVX resistance rate was observed in 92.8% of the isolates. The changes at positions 87 and 91 of the *gyrA* gene were found in isolates with low and high levels of LVX resistance. Two of 5 mutations (Asn87Lys and Asp91Gly) were the most prevalent and the most commonly described in **Table 1**. The results of this study conclude that *H. pylori* resistance usually develops from pre-existing susceptible strains rather than co-infections with different strains (5). The RAPD-PCR has a high discriminatory power that determines the inter- and intra-patient variation of *H. pylori* strains (5).

The application of gene chip technology in *H. pylori* antibiotic resistance detection in children has been reported (73). This technology quick to detect and characterize *H. pylori* infection and the common mutation of genes in multiple resistant sites for CLR (23S rRNA), LVX (GyrA), AMX (PBP1), and TET (16S rRNA), correlating well with the sequencing results (59, 73). Using the gene chip for LVX, a study in China found the *gyrA* gene mutation rate at 87 and 91 loci was 41.7% and 58.3% respectively (73). However, there are no studies reported in Latin America using this technology. Therefore, DNA chips are effective tools for genotyping *H. pylori* in single nucleotide polymorphisms (SNPs) and mutations in the resistance genes simultaneously (73).

Based on the molecular tools, high LVX resistance rates have been reported in countries such as Senegal (81.2%), China, South Korea, and Japan (15.0 to 64.4%), Nepal (42.9%), and Malaysia (25.4%) (45, 65, 67, 71–73). Similarly, the prevalence is highest in Latin American countries such as Colombia from 12.0% to 100% (7, 11, 25, 29), 92.8% in Argentina (5), 47.0% in Venezuela (30), and 39.9% in Ecuador (26), as shown in **Table 1**. When comparing frequency results between molecular and phenotypic methods, there was frequency agreement in 3 of 5 studies (**Table 1**). Two studies showed that the frequency percentage was higher in the genotypic tool than in the phenotypic test. These molecular results detected more mutations in the QRDR region that are not specific to resistant isolates (5). For that reason, it is important to conduct studies in different geographical areas that allow identifying new responsible mutations of fluoroquinolone resistance and update the molecular tools (29).

## Tetracycline (TET)

TETs act on the 30S subunit of the ribosome blocking the binding of aminoacyl-tRNA and discontinuing in protein biosynthesis (6, 74). The TET resistance is due to diverse mechanisms such as membrane-associated efflux proteins that decrease the intracellular concentration of TET; decrease the affinity of ribosomes for TET through ribosomal protection proteins, and single mutations in three contiguous nucleotides of the 16S *rRNA* gene that affect the binding site of TET, located at positions (AGA_926–928_TTC) (74, 75). *In vitro* experiments demonstrated that only the triple mutation leads to high levels of TET resistance whereas in one or two mutations the resistance is low level (57). The absence of a triple mutation may explain the tendency to low or a reduced rate of TET resistance worldwide (64, 74, 76–78). Most *H. pylori* strains are susceptible to TET, an antibiotic commonly used for the eradication of *H. pylori* (74, 79).

The molecular tools mainly used to detect the *H. pylori* TET resistance are PCR and sequencing, RFLP-PCR, RAPD-PCR, and qPCR (6, 74, 80–82). The first study that described the molecular mechanism of TET resistance in an *H. pylori* isolate (strain 181) used PCR y sequencing. Sequence analysis in both copies of the 16S *rRNA* genes revealed that a single triple mutation (AGA_926–928_TTC) was present in a 361 bp PCR fragment and was responsible for TET resistance in *H. pylori* (74).

The PCR-RFLP assay with the restriction enzyme *HinfI*, followed by the DNA sequencing of a fragment (535 bp) of the 16S *rRNA* gene in 41 *H. pylori* Chilean isolates (81). This assay revealed that 11 (27.0%) out of 41 Chilean clinical isolates showed a low-level TET resistance with 1 bp (A928C) or 2 bp (AG926 927→GT and/or A926G/A928C) mutations in both 16S *rRNA* genes. This study determined that the PCR-RFLP assay with DNA sequencing for the accurate detection of TET-resistant *H. pylori* clinical isolates (32). In addition, another study performed in Brazil investigated the high-level TET resistance using PCR-RFLP from *H. pylori*-positive patients (31). *HinfI* RFLP revealed the absence of the AGA_926–928_TTC genotype, indicating that the *H. pylori* high-level TET-resistant was absent in the studied population. It suggests that other genetic factors may explain the TET resistance in gastric samples (31).

Recently, a study from Venezuela used the qPCR to detect the 16S *rRNA* gene mutations related with TET resistance in 96 *H. pylori* isolates (48 obtained from antrum, and 48 from esophagus) (33). The qPCR found single mutations (AGA_926-928_GGA) in six (46.2%) antrum isolates and seven (53.8%) esophagus isolates. Of the 13 isolates with a single mutation, only three were found TET resistant. These results confirm that qPCR is a good tool for distinguishing among TET-resistant genotypes (33). Similarly, another study in Ecuador found that the main mutation with a single base pair substitution was AGA_926–928_GGA (60.0%), while the mutations AGA_926–928_GTA, AGA_926–928_AGC and AGA_926–928_AGT were less prevalent (26). These mutations are associated with low level TET resistance and were found in the Chilean and Venezuelan isolates (32, 33). A recent study by Guzman et al. (7) based on *H. pylori* whole genome sequences from Colombia found a TET resistance rate of 7.23% (12/166) and it demonstrated that the single mutations (A926G, A926T, and A928C) in the 16S *rRNA* gene were related also with low-level TET resistance, as reported in previous studies (31–33). However, the triplet mutation AGA_926–928_TTC associated with a high-level TET resistance has been not found in the studies reported in Latin America.

TET resistance variable rates have been found in Latin America studies from 0% to 85.7% reported by different phenotypic tests (4, 12, 40, 44). Conversely, molecular tools for detecting TET resistance in *H. pylori* offer an alternative to phenotypic tests. The RFLP-PCR, qPCR, and sequencing made it possible to determine TET resistance rates in a range of 0% to 27.0%. Only 2 studies compared resistance results between molecular tools and phenotypic tests, observing that the percentage of resistance coincides between both methods. However, few studies have been carried out in Latin America with these featured techniques, and are shown in **Table 1**. Using qPCR and sequencing, low rates of TET resistance have been reported in Spain (0.2%) (80), and Congo (2.5%) (81), and high rates by RAPD-PCR and DNA sequencing in the US, Canada, Korea, and Japan (54%) (82). Consistently, *H. pylori* TET resistance varies geographically in both phenotypic and molecular methods.

## Metronidazole (MTZ)

MTZ, a synthetic nitroimidazole, acts to damage the DNA helical structure and its mechanism has been described in anaerobic bacteria (83–85).

*H. pylori* MTZ resistance is due to multiple mutations of *rdxA* (encoding for an oxygen-insensitive NADPH nitroreductase), *frxA* (encoding the NADPH flavin oxidoreductase), and *fdxB* (encoding the ferredoxin-like protein) genes (84, 85). However, some resistant strains have not shown mutations in the genes, suggesting that the resistance mechanism involves more complex metabolic changes than those associated with the mutations alone (86).

Resistance is detected in molecular tools such as PCR, RFLP, AS-PCR, qPCR, sequencing, and FISH. Therefore, they can be performed on DNA gastric biopsy or stool specimens, and allow the rapid detection of resistance (73). In Chile, MTZ resistance frequency in the gastric mucosa was 50.0% using PCR and sequencing of the *rdxA* gene obtained from 28 CLR-resistant samples (22). Multiple *rdxA* substitutions were found in MTZ-resistant and sensitive strains. Only 14 (50%) samples showed resistance to MTZ, 8 (57.2%) samples with 5 changes (Arg16His, Ala67Val, Ser88Pro, and Gly162Arg), 1 (7.1%) sample with the Glu75Gln mutation, 1 (7.1%) sample with the Pro166Ala mutation, 1 (7.1%) sample with the Pro166Ser mutation, 2 (14.3%) carried truncating mutations, and one (7.1%) had an in-frame deletion in *rdxA*. These results suggest inactive and missense mutations in *rdxA* give MTZ resistance (22). A study in Bogota, Colombia determined antibiotic resistance in *H. pylori*-positive patients previously treated. This descriptive study with 5 patients showed that 4 were resistant to MTZ (four isolates from the corpus and three isolates from the antrum) and the mutation related to MTZ resistance was Arg90Lys in the *rdxA* gen (87.5%) (11). However, another study in Popayán, Colombia found a high frequency of *rdxA* mutations in 78.0% (34). The most common single mutations were in positions Asp59Asn, Arg131Lys, Arg90Lys, Ala118Thr, Ile160Phe and His97Thr and stop codons Gln50*, Asp59*, Glu75*, Cys159* and Ile160*. All mutations correlate with MTZ resistance, in particular, when there are high levels of MICs (34). Similarly, another study on single mutations associated with MTZ resistance in 166 *H. pylori* isolates from Colombia found a high resistance rate. Single nucleotide polymorphisms in *rdxA* were 99.3% Asp59Asn, 43.3% Arg131Lys, 46.7% Arg90Lys, 26.1% His97Thr, His97Tyr and His97Ile, and 24.8% Ala118Thr and Ala118Ser (7). These results confirm the high variability of MTZ resistance in several regions of Colombia, as described by phenotypic methods (66 to 83%), suggesting not to choose MTZ in eradication therapies by *H. pylori* in Colombia (7, 12). Another study conducted in Cuba analyzed the sequence of the *frx*A gene in *H. pylori*-positive isolates and showed a high frequency of mutations producing stop codons that also increased the levels of MICs, resulting in a MTZ resistance of 56.8% and reaffirming the need for evaluation of the efficacy of this antibiotic in the *H. pylori* therapy (35). In addition, an Ecuadorian study found different genomic changes in the *rdxA* gene from MTZ-resistant and some MTZ-sensitive strains (26). Among amino acid substitutions detected were Met56Ile, Arg16His, Gln50Stop, Leu62Val, Ala118Thr, Met56Val, Gly98Ser, His97Thr, Ser88Pro, His97Tyr, Arg16Cys, Ala68Val, and Ala183Val. However, those mutations probably do not confer resistance to MTZ. All 238 (100%) *H. pylori*-positive samples showed the Asp59Asn mutation, 135 (57.0%) samples the Arg131Lys mutation and 125 (53.0%) samples the Arg90Lys mutation. These multiple mutations were found both in MTZ-resistant and susceptible strains, demonstrating the polymorphisms in *rdxA* and *frxA* association with MTZ resistance. Therefore, the genotypic tools did not apply to MTZ resistance (26).

The MTZ primary resistance data in Latin America were 53.0% by phenotypic methods (12). Similarly, the presence of multiple mutations in the *rdxA* and *frxA* genes varies from 25.0% to 100% both in Latin America and other countries such as Egypt and Nepal (61, 72, 87). Only one study in Latin America compared resistance results between molecular methods and phenotypic tests finding concordance in both methods. However, this study evaluated few isolates (11). In Latin America, like worldwide, molecular detection is not applicable for MTZ due to the variability of mutations found in one of the *rdxA* and *frxA* genes. Finally, the high variability of mutations observed in both resistant and sensitive strains to MTZ, it is recommended to use phenotypic tests to confirm its resistance.

## Discussion

Molecular tools have the advantage of providing rapid, reproducible, and reliable results, and these are useful for routine clinical practice because they can analyze DNA from different samples rapidly, allowing patients to receive appropriate treatment. Likewise, molecular tools detect *H. pylori* mixed infections with multiple genotypes. It is often difficult to culture *H. pylori* because it does not always recover all the strains in the sample (25). An important consideration of molecular studies limitations is the existence of diverse resistance mechanisms to antibiotics in *H. pylori* (16). It involves longer standardization and only detects mutations currently known to confer resistance. For this reason, the details of molecular tools may need to be adapted under the mutations found in each country (25).

All the reviewed studies in Latin America through different molecular tools showed the mutation-associated resistance rates varied from 2.2% to 83.3% for CLA, 12.0% to 100% for LVX, 50.0% to 100% for MTZ, and 0% to 27% for TET and AMX. According to a previously published meta-analysis in Latin America in 2014 using phenotypic tests and PCR (12), resistance rates were lower for CLR, LVX, and MTZ, and higher for TET and AMX than those found with molecular tools. This demonstrates high heterogeneity among studies made in Latin America with different tools. Unfortunately, there is no consensus in the data obtained for each study to explain this variability in the results.

The multi-drug resistance phenomenon in *H. pylori* is a global problem wherefore antibiotics fail in the treatment, and the symptoms and the *H. pylori* infection persist (5, 7). In Latin America, only 3 countries (2 in Colombia, 1 in Argentina and 1 in Chile) reported resistance to two, three, or four antibiotics using molecular tools in a percentage of 33 to 80% (5, 7, 11, 22). Those results have shown an increased multi-resistance rate compared with phenotypic tests reported in 2014 for CLR, LVX and MTZ (12). However, few studies evaluated multi-resistance using molecular tools, suggesting further research to confirm these estimates.

Cost, locally available equipment, and experience in molecular techniques are factors that influence the practicality of molecular tools in laboratories from Latin America (88). Although the cost of the molecular tools is higher than conventional phenotypic tests, their sensitivity and specificity are higher. The use of molecular tools reduces the time for confirmation of the detection of multi-drug resistant *H. pylori* infection, allowing the adaptation of the treatment in a shorter time.

## Conclusions

Molecular studies from nine Latin American countries have provided valuable insights into *H. pylori* resistance to antibiotics. Molecular tools used for CLR, LVX, and TET are useful for the rapid detection of resistance without the need for culture. Whereas, resistance for MTZ and AMX could not be evaluated by genotypic tools, because exist different mutations in both resistant and sensible strains and there is no consensus to define which mutations are responsible for resistance in these antibiotics.

The revision of the different molecular methods showed that qPCR and RFLP are the best tools to detect the resistance of *H. pylori*. These methods are faster, low-cost test that avoids reading errors, allowing analysis of different samples like gastroesophageal mucosa, feces, or gastric juice, representing an excellent option compared with culture and phenotypic tests. Accurate molecular tools can significantly improve the selection of effective treatments in clinical practice, emphasizing the importance of continued research in this field.

## Author contributions

MC: Conceptualization, Writing – original draft, Writing – review & editing, Data curation, Investigation. HM: Conceptualization, Writing – original draft. MG-A: Conceptualization, Writing – review & editing, Writing – original draft.
